# Palisade cell shape affects the light-induced chloroplast movements and leaf photosynthesis

**DOI:** 10.1038/s41598-018-19896-9

**Published:** 2018-01-24

**Authors:** Eiji Gotoh, Noriyuki Suetsugu, Takeshi Higa, Tomonao Matsushita, Hirokazu Tsukaya, Masamitsu Wada

**Affiliations:** 1 Faculty of Agriculture, Kyushu University, Fukuoka, 812-8581 Japan; 2 Graduate School of Biostudies, Kyoto University, Kyoto, 606-8502 Japan; 3 Institute of Molecular, Cell, and Systems Biology, College of Medical, Veterinary, and Life Sciences, University of Glasgow, Glasgow, G12 8QQ United Kingdom; 4 Institute for Protein Research, Osaka University, Suita, Osaka, 565-0871 Japan; 5 Department of Biological Sciences, Graduate School of Science, The University of Tokyo, Tokyo, 113-0033 Japan; 6 Department of Biological Sciences, Graduate School of Science and Engineering, Tokyo Metropolitan University, Tokyo, 192-0397 Japan

## Abstract

Leaf photosynthesis is regulated by multiple factors that help the plant to adapt to fluctuating light conditions. Leaves of sun-light-grown plants are thicker and contain more columnar palisade cells than those of shade-grown plants. Light-induced chloroplast movements are also essential for efficient leaf photosynthesis and facilitate efficient light utilization in leaf cells. Previous studies have demonstrated that leaves of most of the sun-grown plants exhibited no or very weak chloroplast movements and could accomplish efficient photosynthesis under strong light. To examine the relationship between palisade cell shape, chloroplast movement and distribution, and leaf photosynthesis, we used an *Arabidopsis thaliana* mutant, *angustifolia* (*an*), which has thick leaves that contain columnar palisade cells similar to those in the sun-grown plants. In the highly columnar cells of *an* mutant leaves, chloroplast movements were restricted. Nevertheless, under white light condition (at 120 µmol m^−2^ s^−1^), the *an* mutant plants showed higher chlorophyll content per unit leaf area and, thus, higher light absorption by the leaves than the wild type, which resulted in enhanced photosynthesis per unit leaf area. Our findings indicate that coordinated regulation of leaf cell shape and chloroplast movement according to the light conditions is pivotal for efficient leaf photosynthesis.

## Introduction

The blue-light (BL) receptor, phototropin (phot), regulates phototropism, chloroplast movement, stomatal opening, and leaf movement and development, all of which ensure efficient light utilization for photosynthesis^1,2^. Light-induced chloroplast movement (hereafter, referred to as “chloroplast movement”) is found in various plant species, including algae and land plants^3^. Under low light conditions, chloroplasts move toward light-irradiated area (the “accumulation response”) and are situated on the periclinal cell walls, ensuring the capture of maximum amount of light. Conversely, chloroplasts escape from strong light (the “avoidance response”) and consequently localize on the anticlinal cell walls where light absorption is low. The avoidance response is essential for avoiding the photodamage and for survival under the natural strong light conditions^4^. The avoidance response is also implicated in facilitating the penetration of light into deeper cells and diffusion of CO_2_ from air spaces into the mesophyll chloroplasts as a result of increase in the surface area of chloroplasts exposed to intracellular air spaces^5^.

Chloroplast movement can be detected by measuring the light-induced changes in leaf transmittance (or absorption)^6,7^. A decrease in leaf transmittance represents the chloroplast accumulation response and an increase reflects the avoidance response. This method of detection is easy and non-invasive and has been used to analyze chloroplast movements in various plant species, including flowering plants, ferns, and mosses^8–16^. Although the magnitude of chloroplast movement varies among the plant species, shade-grown plants tend to exhibit stronger chloroplast movement than sun-grown plants^8–11,13,16^. However, some ferns that can grow under a wide range of light conditions have been observed to exhibit stronger chloroplast movement than those that grow under limited light conditions or in a shade^12^. Notably, no or very subtle chloroplast movements were detected in some sun-grown plants, including climbing plant species^8,13,16^. In general, the mesophyll cells in palisade cell layers are spherical in the leaves of shade-grown plants. Leaves of sun-grown plants are thicker than those of shade-grown plants. Because the palisade mesophyll cells are more columnar, one or more tiers of columnar palisade cells could facilitate penetration of light into deeper cell layers^5,17,18^. The more columnar cells in the sun-grown leaves restrict the chloroplast movements and most of the chloroplasts remained aligned on the anticlinal walls regardless of the light conditions^5,13,16^. The sun-grown leaves should contain more cells and, thus, more chloroplasts per unit leaf area, because total plasma membrane area of the columnar cells per unit leaf area would be larger than that of the spherical cells in the shade-grown plants^16^. Therefore, the presence of more columnar cells in the sun-grown leaf could contribute to the higher photosynthetic performance per unit leaf area. The constitutive positioning of chloroplasts on the anticlinal walls could be beneficial under strong light for leaf photosynthesis by facilitating the penetration of light into deeper cell layers^5,17,18^ or CO_2_ diffusion^5,19^.

To substantiate the fact that chloroplast movements are restricted in the columnar palisade cells, we analyzed the relationship between the shape of palisade cells and chloroplast movement in the same plant species grown under the same light condition. We used *Arabidopsis thaliana angustifolia* (*an*) mutant plants^20^. The *an* mutant plants exhibit narrower and thicker leaves although the length of the leaf blade is similar to those of the wild-type (WT) plants. This leaf phenotype in *an* mutants is caused by the reduction in the size of palisade cells in the direction of leaf width, accompanied with an increase in cell size in the direction of leaf thickness, indicating that the palisade cells in the *an* mutants are more columnar than those in the WT^20^. However, the total number of cells in the leaves of WT and *an* mutant plants is similar^20^. Thus, the cell structure in the *an* mutant leaves mimics that of sun-grown leaves, except for the narrow leaf width. As a control for the narrow leaf mutant, we used another narrow leaf mutant, *an3*^21^. The *an3* mutant leaves look like the *an* mutant leaves, but the narrow leaf phenotype in *an3* is attributable to the severe reduction in the number of cells in the leaves^21^. The size of cells in leaves is larger in *an3* mutants compared to that in the WT^21^. Here, we compared leaf photosynthesis and chloroplast movements between WT, *an*, and *an3* mutant plants.

## Results

### Leaves of *an* mutant plants have several characteristics similar to those of sun-grown-leaves

**Figure 1:**
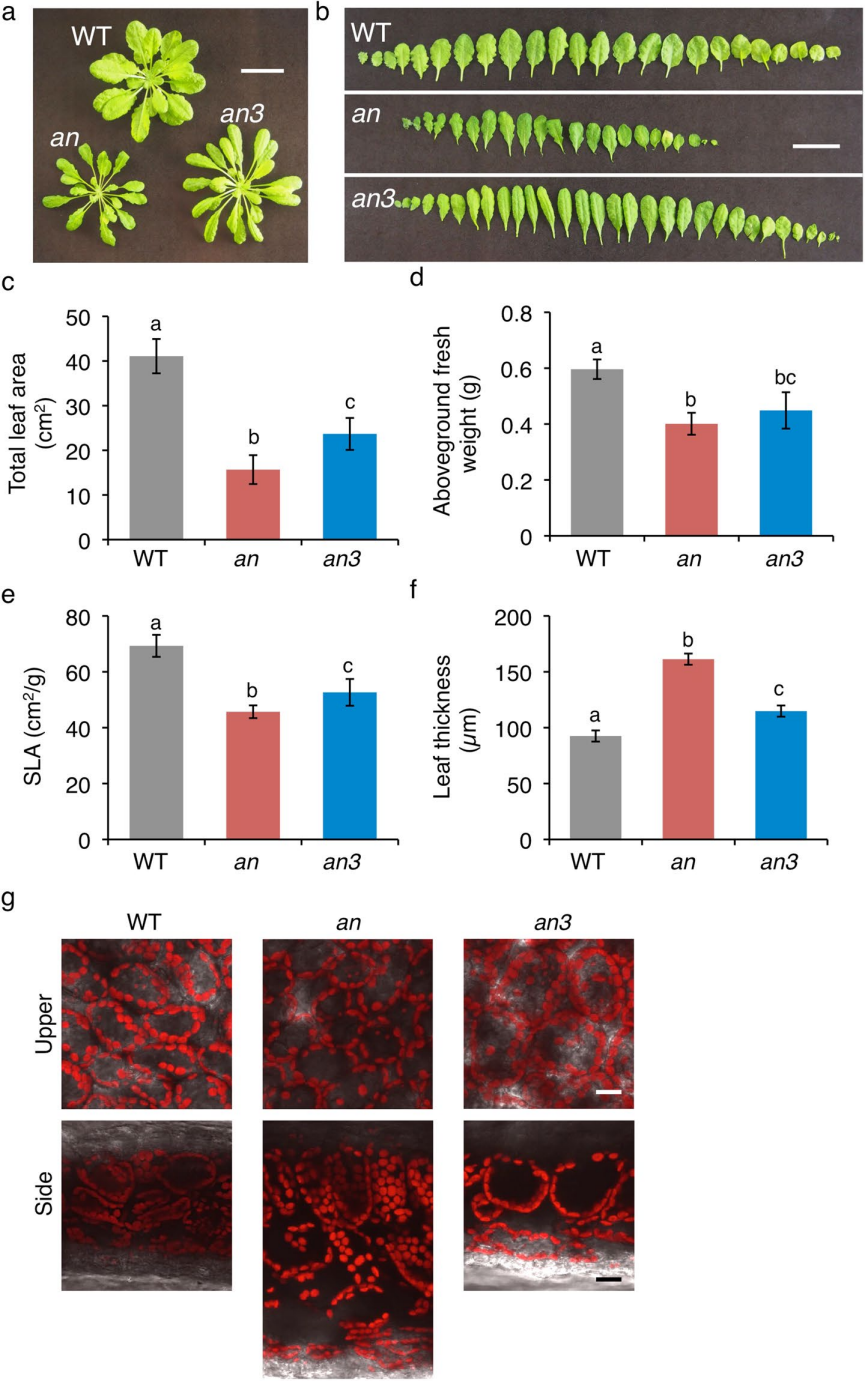
Altered leaf morphology in *an* and *an3* mutants. (**a**) Photograph of 42-day-old plants. Scale bar = 2 cm. (**b**) Photographs of leaves detached from 42-day-old plants. The left leaf is the youngest and the right is the oldest one (it is one of the cotyledons). Scale bar = 2 cm. (**c**–**e**) Total leaf area (**c**), aboveground fresh weight (**d**), and specific leaf area (SLA, projected leaf area per unit leaf fresh weight: total leaf area/aboveground fresh weight) (**e**) of 42-day-old wild-type (WT) and mutant plants. Data show the mean ± SEM (*n* = 24) of three independent experiments. Significant differences (*P* < 0.05, Tukey-Kramer) are indicated by different characters. (**f**) Thickness of leaves in the WT and mutant plants. Data show the mean ± SEM (*n* = 9) of three independent experiments. Significant differences (*P* < 0.05, Tukey-Kramer) are indicated by different characters. (**g**) Leaf cell morphology and chloroplast distribution in *an* and *an3* mutants. Wild type (WT) and mutant plants were grown under white light condition (120 µmol m^−2^ s^−1^) for 42 days. Views of the upper surface of palisade tissue cells (upper panel) and cross sections (lower panel) of the leaves from the WT and the mutant plants are shown. Scale bar = 20 µm.

When WT, *an*, and *an3* mutant plants were grown under white light condition (at 120 µmol m^−2^ s^−1^), the rosette size was similar in the WT and *an3* mutant plants, but was smaller in the *an* mutant plants (Fig. 1a). Both the *an* and *an3* mutant leaves were narrower than the WT leaves (Fig. 1a and b), as described previously^20,21^. Consistently, the total leaf area and leaf weight were smaller in the *an* and *an3* mutants compared to that in the WT plants (Fig. 1c and d). The value of the specific leaf area (SLA), which is the ratio of total leaf area to the fresh weight, in both the *an* and *an3* plants was small (Fig. 1e). The lower values of SLA generally mean that the leaves are thicker. Indeed, the leaves of *an* mutant plants were much thicker than those of WT (Fig. 1f and g), as described previously^20^. The *an3* leaves were also thicker than the WT leaves although they were thinner than the *an* leaves (Fig. 1f). Compared to the WT leaves, the periclinal cell size of the first palisade cells was slightly smaller, but the anticlinal cell size was much larger in the *an* leaves (Fig. 1g and Table 1). The *an* palisade cells were much longer in the direction of leaf thickness (Fig. 1g and Table 1) and, thus, displayed a columnar shape, as described previously^20^. Consistent with the previous results^21^, the palisade cells of the *an3* leaves had larger cell size at both the periclinal and anticlinal sides (Fig. 1g and Table 1). The length of palisade cells in the *an3* leaves in the direction of leaf thickness was intermediate between the lengths in the WT and *an* leaves (Fig. 1g and Table 1). Under the growth conditions used in this study, the *an* mutant leaves often contained two layers of the palisade cells, but the WT and *an3* leaves contained only one layer (Fig. 1g). Therefore, the *an* mutant leaves are somewhat similar to the sun-grown leaves in that they are thick and have columnar palisade cells, although the *an3* mutant leaves are also thick but have less columnar cells.

**Table 1:** Palisade cell and chloroplast size in wild type (WT), *an*, and *an3* mutant plants.

	Cell						Chloroplast				
	Periclinal area (μm^2^)		Cell length (μm)		Anticlinal wall area (μm^2^)		Surface area (μm^2^)		Number/Cell		Total area/Cell (μm^2^)
WT	1515.4	±123.9	32.6	±2.2	3360.6	±267.2	39.1	±1.4	51.0	±1.7	1997.9
*an*	1236.6	±11.4	59.2	±5.0	5708.0	±640.0	43.9	±4.1	64.1	±1.4	2812.0
*an3*	2223.2	±182.5	45.3	±3.9	6203.7	±454.3	34.3	±0.9	74.1	±4.5	2541.6

### Photosynthesis per unit leaf area is enhanced in *an* and *an3* mutant leaves

**Figure 2:**
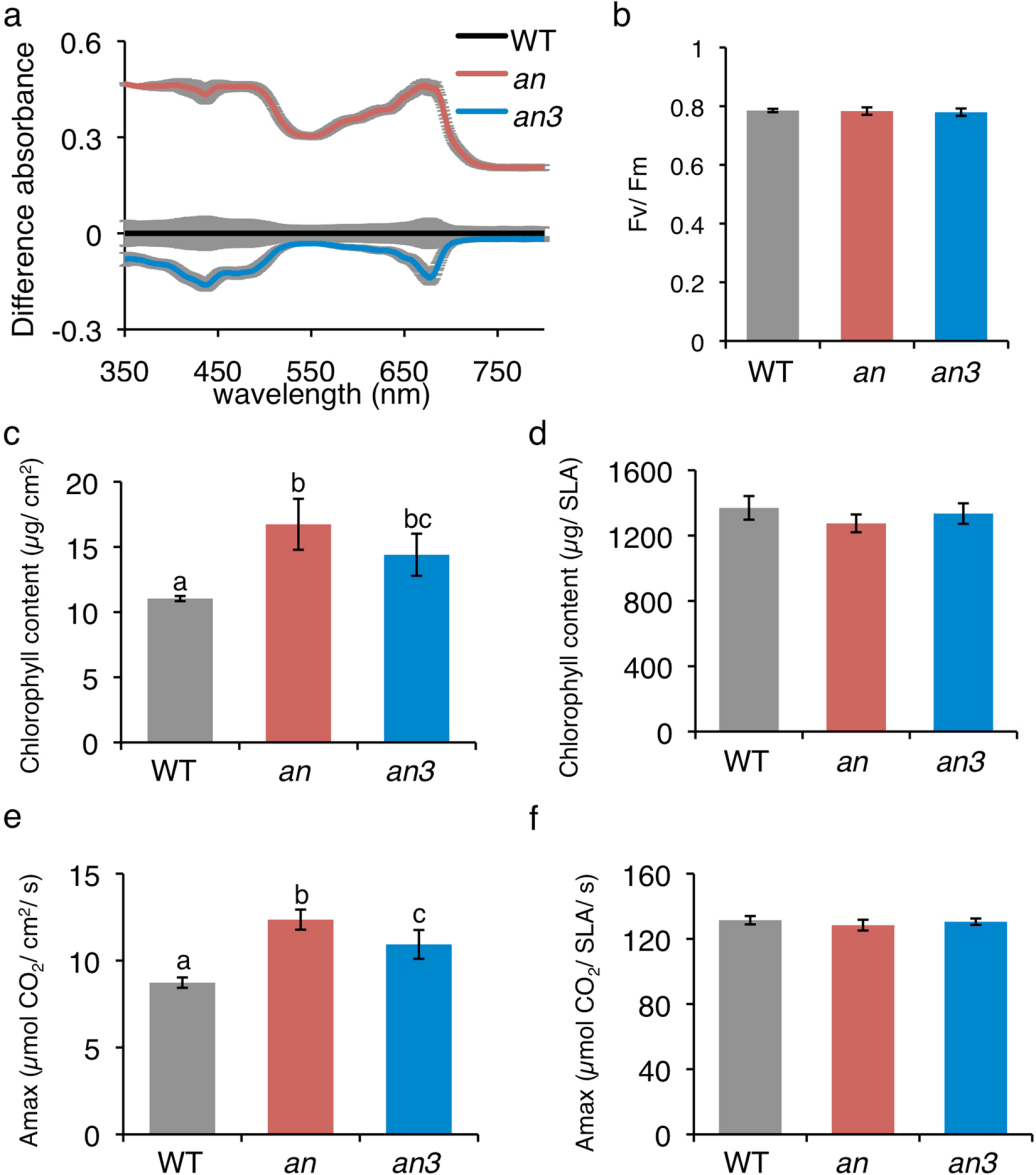
Photosynthetic performance of *an* and *an3* mutants. (**a**) Difference spectra of leaf absorbance between the wild-type (WT) and mutant plants. Leaf absorbance was measured under white light (120 µmol m^−2^ s^−1^). The difference in multi-wavelength (350–800 nm) absorbance was calculated by subtracting the absorbance of each mutant from that of the WT. Data show the mean ± SEM of three independent experiments. (**b**) Maximum photochemical efficiency of PSII (Fv/ Fm) of the leaves in WT, *an*, and *an3* mutant plants. After keeping the leaves in dark for at least 1 h, Fv/Fm was measured. Data show the mean ± SEM of three independent experiments. (**c**–**f**) Chlorophyll content and photosynthetic capacity. (**c** and **d**) Total chlorophyll content of leaves in the WT and mutant plants. The chlorophyll content of rosette leaves of 42-day-old plants was determined. (**e** and **f**) Maximum CO_2_ assimilation capacity (Amax) in WT, *an*, and *an3* mutant plants. Amax was calculated from each light saturation point. The chlorophyll content and photosynthetic capacity are expressed per leaf area (**c** and **e**) or per SLA (**d** and **f**). SLA was calculated using detached leaves. Data show the mean ± SEM of three independent experiments.

Consistent with the presence of thicker leaves in the *an* mutant plants, the light absorbance by these leaves was much higher than in the WT (Fig. 2a). However, the light absorbance by the leaves of the *an3* mutant plants was slightly lower than that in the WT plants (Fig. 2a). Thus, photosynthetic light utilization could be different between the WT and mutant plants. Under the growth conditions used in this study, the maximum quantum yield of photosystem II (PSII), Fv/Fm, was normal in all the lines (Fig. 2b), indicating that the *an* and *an3* mutant plants had no detectable defects in the electron transport around PSII and there was no damage to the PSII under the experimental conditions. Consistently, the levels of ribulose-1,5-bisphosphate carboxylase/oxygenase large subunit (RbcL), PsaA (a core protein of photosystem I), PsbB (CP47 protein of photosystem II), cytochrome *f* protein of the thylakoid Cyt b6/f-complex (Cyt *f*), and plastocyanin (PC) did not differ between the WT and mutant plants (Supplemental Figs 1 and 2). However, differences were observed in the chlorophyll content and maximum CO_2_ assimilation rates between the WT and the mutant plants (Fig. 2c to f). When the chlorophyll a and b contents were measured on the basis of unit leaf area, there was no difference in the chlorophyll a/b ratio (the values of mean ± SEM for WT, *an*, and *an3* were 2.32 ± 0.36, 2.43 ± 0.23, and 2.69 ± 0.10, respectively). However, the values of total chlorophyll content per leaf area were higher in the *an* mutant plants (Fig. 2c). Concomitant with the higher chlorophyll contents, the CO_2_ assimilation rate per leaf area was much higher in the *an* mutant plants (Fig. 2e). Unexpectedly, *an3* mutants also exhibited higher chlorophyll contents and CO_2_ assimilation rate per leaf area although the values were lower than those in the *an* mutant plants (Fig. 2e). Importantly, the values of total chlorophyll content and CO_2_ assimilation rate per SLA were comparable between the WT and mutant plants (Fig. 2d and f). Therefore, the enhanced photosynthesis per leaf area in the *an* and *an3* mutants should be attributable to the thicker leaves and/or the altered structure of leaf cells.

### Light-induced chloroplast movements are restricted in *an* mutants but not in *an3* mutants

**Figure 3:**
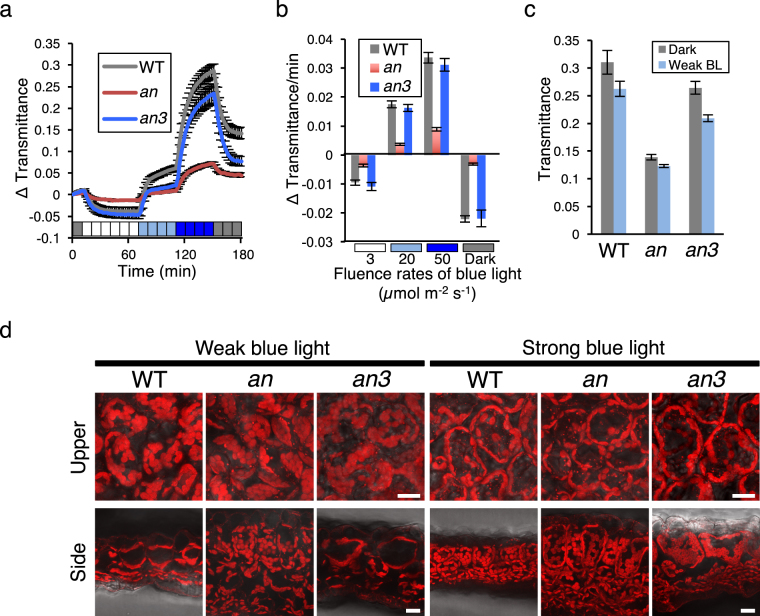
Light-induced chloroplast movements and intracellular distribution in *an* and *an3* mutants. (**a**) Changes in leaf transmittance caused by light-induced chloroplast movements in wild-type (WT), *an*, and *an3* mutant plants. Coloured boxes on the horizontal axis indicate passage of time (one box denotes 10 min) and light conditions. After 10 min exposure to darkness (indicated by black boxes), the leaves were sequentially irradiated with blue light (BL) at 3, 20, and 50 µmol m^−2^ s^−1^ for 60, 40, and 40 min (indicated by white, sky blue, and blue boxes, respectively). The light was turned off at 150 min. (**b**) The average of the changes in transmittance over 1 min was calculated by measuring the changes in the leaf transmittance rates for 2–6 min after changes in the light fluence rates (3, 20, and 50 µmol m^−2^ s^−1^ or dark). (**c**) Leaf transmittance at 0 and 70 min (i.e., 60 min after weak BL irradiation) after the onset of measurement of leaf transmittance changes. Data in a to c show the mean ± SEM of three independent experiments. (**d**) Chloroplast distribution in the WT and mutant plants irradiated with weak BL (3 µmol m^−2^ s^−1^) and strong BL (50 µmol m^−2^ s^−1^) for 3 h. Views of the upper surface of palisade tissue cells (upper panel) and cross sections (lower panel) of leaves from the WT and mutant plants are shown. Scale bar = 20 µm.

The BL-induced chloroplast movements were analyzed by measuring the light-induced changes in leaf transmittance^22^. In WT plants, weak BL (3 µmol m^−2^ s^−1^) induced a decrease in leaf transmittance as a result of the chloroplast accumulation response whereas an increase in the leaf transmittance was induced by strong BL (20 and 50 µmol m^−2^ s^−1^) as a result of the avoidance response (Fig. 3a and b). After the strong blue light was turned off, a rapid decrease in leaf transmittance was induced (which is referred to as the “dark recovery response”)^23^. The chloroplast movements were almost normal in the *an3* mutant plants (Fig. 3a). The speed (the average of the changes in transmittance over 1 min for 2–6 min after changes in the light fluence rates) of accumulation, avoidance, and dark recovery responses were not significantly different from those in WT (Fig. 3b; one-way ANOVA followed by Tukey–Kramer multiple comparison *post hoc* test, *P* > 0.5 in all the light treatments), although the amplitude of the avoidance response at 20 µmol m^−2^ s^−1^ was smaller in the *an3* mutant plants (Fig. 3a). Conversely, in the *an* mutant plants, the light-induced changes in leaf transmittance were severely attenuated (Fig. 3a). The accumulation, avoidance, and dark recovery responses were detectable, but both the speed and amplitude of these responses were strongly suppressed in the *an* mutant plants (Fig. 3a and b; one-way ANOVA followed by Tukey–Kramer multiple comparison *post hoc* test, *P* < 0.01 in all the light treatments). The leaf transmittance before and after the weak BL irradiation was much lower in the *an* mutant plants than in the WT, consistent with thicker leaves in the *an* mutant plants (Fig. 3c; one-way ANOVA followed by Tukey–Kramer multiple comparison *post hoc* test, *P* < 0.01). Therefore, these results indicate that the shape of palisade cells, but not the cell volume, is an important factor in the restriction of chloroplast movements.

To examine the distribution of chloroplasts in the low- or high-light-irradiated WT and mutant plants (Fig. 3d), the number and plan area of chloroplasts on both the periclinal and anticlinal walls was measured and the rate in terms of the number or area that the chloroplasts occupied on the periclinal or anticlinal walls (the “occupancy rates of chloroplast number” or “occupancy rates of chloroplast area”, respectively; see Methods for details) was calculated (Table 2)^16^. In all the lines, both the accumulation and avoidance responses were induced in the low- and high-light-irradiated plants, respectively (Fig. 3d), but the occupancy rates of chloroplast number and area were different between the WT and mutant plants (Table 2). On the periclinal walls of the *an3* palisade cells, the rate of chloroplast number was higher than that in the WT, irrespective of the light conditions, and the rate of chloroplast area was higher in the high light-irradiated plants, but was similar to that of the WT in the low light-irradiated plants. The rate of chloroplast area on the anticlinal walls was also lower in the *an3* mutant plants, irrespective of the light conditions. The occupancy rates, based on chloroplast area, on the periclinal walls in the *an* mutants were significantly lower than those in the WT only under high light conditions. However, under both low and high light conditions, the occupancy rates, according to the number of chloroplasts, on the periclinal walls in the *an* mutants were much lower than those in the WT, indicating that most of the chloroplasts in the *an* mutants resided on the anticlinal walls. Therefore, these results suggest that the chloroplast movement and distribution pattern in the *an* mutant plants are similar to those in the sun-grown plants, including the climbing plant species.

**Table 2:** Chloroplast distribution in the palisade tissue cells under weak and strong blue light.

	Low light (the “accumulation response”)						
	Periclinal				Anticlinal		
	Actual chloroplast number		Occupancy rate of chloroplast number (%)	Occupancy rate of chloroplast area (%)	Calculated chloroplast number	Occupancy rate of chloroplast number (%)	Occupancy rate of chloroplast area (%)
WT	20.1	±1.4	39.4	51.9	30.9	60.6	36.0
*an*	13.5	±1.2	21.1	48.0	50.5	78.9	38.9
*an3*	34.1	±1.3	46.0	52.6	40.0	54.0	22.1
**High light (the “avoidance response”)**							
WT	4.0	±0.1	7.8	10.2	47.1	92.2	54.8
*an*	0.9	±0.2	1.4	3.2	63.2	98.6	48.6
*an3*	10.8	±2.6	14.6	16.7	63.3	85.4	35.0

## Discussion

Previous studies, using various plant species, have led to an assumption that more columnar cells could restrict the chloroplast movement^8–11,13,16^. To test this assumption, we used the *Arabidopsis an* mutant plants as a model system. Our findings indicate that the cell shape determines how easily chloroplasts can move in response to light.

The present and previous analyses of *an* leaves indicate that the *an* leaves are thicker than the WT leaves and the thick leaves result from more columnar cells and more cell layers (Fig. 1 and Table 1)^20^. The *an3* mutant plants are defective in the proliferation of leaf cells and the palisade cells are larger than those of WT^21^, because of “compensation” mechanism in which cell proliferation and expansion are tightly regulated to ensure appropriate organ size^24^. In addition to previous analysis of leaf paradermal sections^21^, our analysis of the leaf cross sections indicates that the *an3* palisade cells are longer in all the directions and, thus, does not show columnar shape in contrast to *an* palisade cells (Fig. 1 and Table 1). Furthermore, the *an3* leaves are thicker than the WT leaves although they are thinner than the *an* leaves. Because there was no increase in the number of cell layers in the *an3* mutants (Figs 1g and 3d), the increase in leaf thickness should have resulted from the larger palisade cells.

The *an* mutant plants showed much higher leaf absorption, chlorophyll content, and photosynthetic performance per unit leaf area (Fig. 2), indicating that thick leaves consisting of columnar cells facilitate photosynthetic light capture and, thus, enhance the photosynthetic performance. In sun-grown plants, especially in climbing plants, most chloroplasts are constitutively localized on the anticlinal walls irrespective of the light conditions^16^. Because the palisade cells are highly columnar, the periclinal area is very small and, thus, the accumulation response is not effective in these plants. The constitutive localization of chloroplasts on the anticlinal walls should facilitate photoprotection under strong light conditions, in the location where climbing plants are living^16^. It should also facilitate penetration of light into the deeper cell layers^5,17,18^. Although it was not prominent, compared to that in the climbing plants, a higher percentage of chloroplasts were localized on the anticlinal walls in the *an* mutant plants (Table 2). Indeed, consistent with previous analysis in sun-grown plants^5,13,16^, the light-induced changes in leaf transmittance was severely attenuated in the *an* mutant plants (Fig. 3). This phenotype in the *an* mutant plants were similar to those in the *plastid movement impaired 1* (*pmi1*) mutant plants^25,26^. The chloroplast movement is dependent on actin filaments^27^ and PMI1 is necessary for the regulation of actin filaments during the light-induced chloroplast movement^26^. However, unlike in the *an* mutant leaves, leaf morphology and transmittance are normal in the *pmi1* mutants^25,26^, indicating that defects in the leaf transmittance change between the *an* and *pmi1* mutant plants are caused by different mechanisms. AN is a plant homolog of CtBP/BARS that functions as a transcriptional corepressor or regulator of membrane trafficking in mammals^28,29^. Although the exact function of plant AN proteins is unknown, the *Arabidopsis* AN protein is implicated in the vesicle trafficking^30^ and post-transcriptional regulation^31^. However, only a small number of genes was derepressed in the non-stressed *an* mutants^28^. Consistently, the phototropin protein level was normal in the *an* mutants (Supplemental Figs 1 and 2). Therefore, it is likely that the reduced light-induced changes in leaf transmittance in *an* mutants could be caused by the altered leaf cell geometry but not by the defects in the molecular mechanism for chloroplast movements. The *an3* mutants exhibited almost normal light-induced changes in leaf transmittance although slightly higher number of chloroplasts still reside on the peliclinal wall under HL conditions (Table 2). The *an3* mutant cells are larger and, thus, have more space for chloroplasts to move than WT and *an* mutant. However, at least in our experimental time scale (i.e., blue light irradiation for 40~60 min), the difference in the light-induced changes in leaf transmittance between WT and *an3* might have not been detected. Nevertheless, *an3* exhibited normal light-induced changes in leaf transmittance although their leaves are thick and the palisade cells are longer in the direction of leaf thickness as in the case of *an* mutants. Therefore, restricted chloroplast movement should be attributable to more columnar cells in the *an* mutants. In more columnar cells, chloroplasts could be appressed to the anticlinal walls, as suggested previously^5^.

In conclusion, the shape of cells in the leaves strongly affects the movement and distribution of chloroplasts. The coordination between the cell shape and chloroplast distribution is essential for efficient leaf photosynthesis and, thus, for the adaptation to ambient light conditions. The thick *an*-like leaves, that have long palisade cells and the greater amount of chloroplasts per unit area, are clearly beneficial to plants that are always exposed to strong light, for example the climbing plants. However, under weak light conditions, cells in the deeper layers can not capture light efficiently and perform efficient photosynthesis there because a large part of light could be used only in the first palisade cell layer in the *an*-like leaves. Importantly, it was shown in multiple plant species, including *Arabidopsis*^32^, that strong light makes palisade cells more columnar. In *A. thaliana*, this light-dependent palisade cell development is regulated in phototropin2- and photosynthesis-dependent manners^32^. Thus, phototropins enhance leaf photosynthesis by regulating cell development as well as chloroplast positioning in leaves.

## Methods

### Plant materials and growth conditions

The *Arabidopsis thaliana* WT, *an* (*an-1*)^20^ and *an3* (*an3-4*)^21^ plants used in this study were in the Columbia-0 background. For growth analysis, leaf anatomy, and photosynthetic measurements, plants were grown in soil in a controlled growth chamber (at 22 °C, 55% relatively humidity, and 8 h day light conditions) under white light at 120 µmol m^−2^ s^−1^. For the measurement of light-induced changes in leaf transmittance, seedlings were cultured on 0.8% agar medium containing 1/3 strength Murashige and Skoog’s salt and 1% sucrose, and grown under white light at ca. ~100 µmol m^−2^ s^−1^ (16 h)/dark (8 h) cycle at 23 °C in an incubator.

### Analysis of spectral light absorbance

For measurements of the spectral light absorbance in a whole leaf, detached leaves from 3-week-old plants were placed on the surface of 1% gellan gum and irradiated with white light at 120 µmol m^−2^ s^−1^ for 3 h. The absorbance at wavelengths ranging from 300 to 800 nm was measured at every 1 nm with a microplate reader (Multiskan GO, ThermoFisher).

### Quantification of plant biomass and leaf thickness

Immediately after the fresh weights of all the rosette leaves of 6-week-old plants were measured, their photographs were taken. Leaf thickness was measured from microscopic images (TCS SP8, Leica) of sections of leaves from the 6-week-old plants. The measurements of leaf area and thickness were carried out with Image J (National Institutes of Health).

### Analysis of CO_2_ assimilation

CO_2_ assimilation in intact leaves was analyzed with an open gas exchange system (Li-6400, LI-COR) attached to a normal chamber (LI-COR). After the plants were dark-adapted for at least 3 h, the measurements were performed under a controlled atmospheric conditions (temperature 22 °C, relative humidity 50–60%, and a CO_2_ concentration of 400 µl L^−1^). The light response curve of photosynthesis was obtained according to the protocol provided by the manufacturer, and was used for determining the saturation value of CO_2_ assimilation. The value of Amax was calculated as the average maximum net photosynthesis.

### Estimation of chlorophyll content

Chlorophylls were extracted from four or five full-expanding leaves with 80% acetone. The OD of the clarified chlorophyll extracts were measured at 645 nm and 663 nm and the chlorophyll content was calculated as described in a previous report^33^.

### Immunoblot analysis

Total protein was extracted from the rosette leaves of 6-week-old plants in a protein extraction buffer that contained 50 mM Tris⋅HCl (pH 7.5), 100 mM NaCl, 5 mM EDTA, 0.5% Triton X-100, 1 mM DTT, and 1 mM PMSF. After SDS-PAGE on 12% acrylamide gels, the proteins were transferred to polyvinylidene fluoride membranes. Antibodies against phot1^34^, phot2^35^, RbcL, PsaA, PsbB, Cyt *f*, and PC (Agrisera, Vännäs, Sweden) were used for western blotting experiments.

### Analyses of chloroplast photorelocation movements

Chloroplast photorelocation movements were analyzed by measuring the light-induced changes in leaf transmittance, as described previously^22^. The third leaves were excised from 16-day-old seedlings and placed on 1% (w/v) gellan gum in a 96-well plate. The leaves were dark-adapted for at least 1 h and were used for the measurement of transmittance.

### Observation of chloroplast distribution patterns

Three-week-old plants were irradiated with weak BL and strong BL for 3 h. The cross-sections of leaves that were fixed with 2.5% glutaraldehyde (WAKO) were made with a vibrating microtome (VT1200 S, Leica). Intracellular chloroplast distribution on the upper cell surface of the palisade cells and in the cross-sections was observed under a laser scanning confocal microscope (TCS SP8, Leica). For confocal microscopic imaging (Figs 1g and 3d), the projection images were constructed from z-stacks using the software supplied by the manufacturer. The number of chloroplasts at the periclinal walls was counted after the weak- or strong-BL irradiation, and was used for calculation of the number of chloroplasts at the anticlinal walls as the difference from the total chloroplast number in a cell shown in Table 1. Data for chloroplast distribution pattern in Table 2 was taken as described previously^16^. Under weak BL irradiation, the occupancy rates of chloroplast number were calculated as the percentage of chloroplasts accumulated to the periclinal walls or remaining at the anticlinal walls compared to the total number of chloroplasts in a cell. The occupancy rates of chloroplast number were calculated as the percentage of chloroplasts that moved toward the anticlinal walls or remained at the periclinal walls compared to the total number of chloroplasts in a cell under strong BL exposure. The occupancy rate of chloroplast area is the percentage of periclinal or anticlinal wall area, which calculated as the projection or surface area and shown in Table 1, occupied by the chloroplast area and multiplied by the number of chloroplasts at the periclinal or anticlinal walls.

### Statistical analysis

Comparisons among the groups were performed by using one-way ANOVA followed by Tukey–Kramer multiple comparison *post hoc* test. The differences were considered to be significant at *P* < 0.05. Statistical analysis was performed using Excel 2011 (Microsoft, USA) with the add-in software Statcel 3^36^.

## Electronic supplementary material

Supplementary information
