# Supplementary material for: Palisade cell shape affects the light-induced chloroplast movements and leaf photosynthesis
Source: Sci Rep. 2018 Jan 24;8:1472. doi: 10.1038/s41598-018-19896-9 (PMC5784166; doi:10.1038/s41598-018-19896-9)

Supplementary Information for

Palisade cell shape affects the light-induced chloroplast movements and leaf photosynthesis

Eiji Gotoh, Noriyuki Suetsugu*, Takeshi Higa, Tomonao Matsushita, Hirokazu Tsukaya and Masamitsu Wada*

*To whom correspondence should be addressed: n.suetsugu@gmail.com and masamitsu.wada@gmail.com

This file contains Supplementary Figures S1 and S2

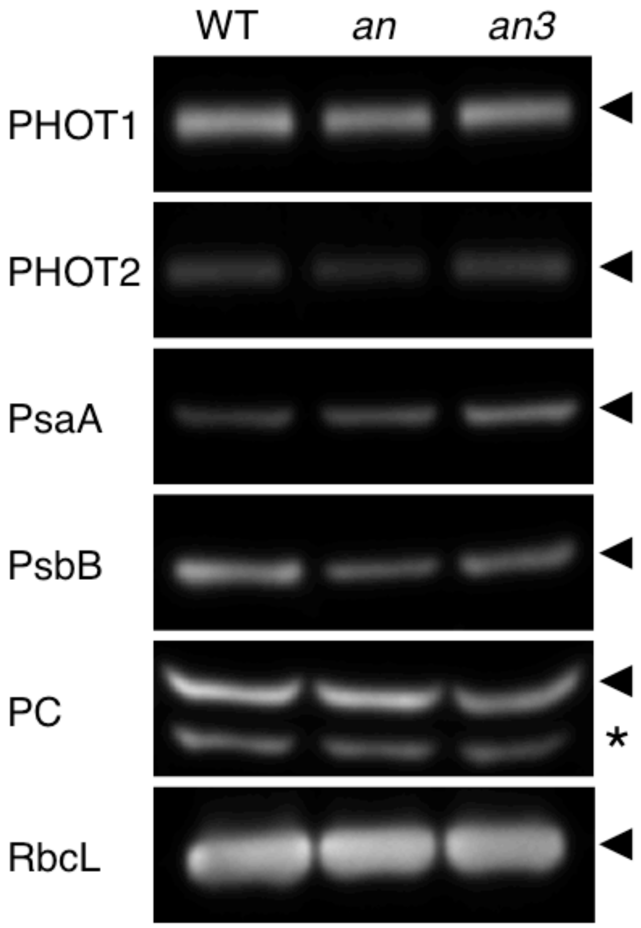

Supplemental Figure 1. Immunoblot analyses of phototropin and photosynthesis-related proteins. Polyclonal antibodies against PHOT1, PHOT2, PsbB, PsaA, plastocyanin (PC), ribulose-1,5-bisphosphate carboxylase/oxygenase (RbcL) were used. For immunoblot analysis of PHOT1, PHOT2, PsaA, PsbB and PC, 20 μg of the total proteins was used and for RbcL, 5 μg of the total proteins was used. Arrows indicate the specific bands. Asterisk indicates a non-specific band. These blots are derived from different gels (see Supplemental Figure 2).

Supplemental Figure 2. Images of full-length blots for Supplemental Figure 1.

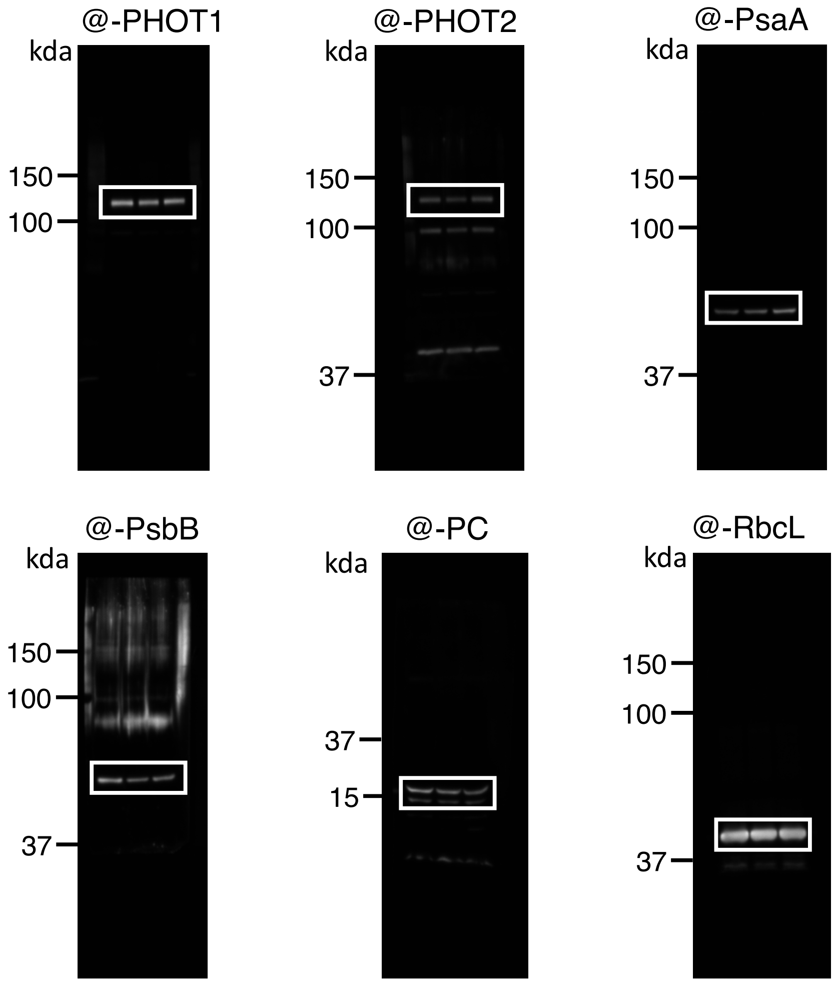

Supplement: Supplementary file 1 — Supplementary information [file 41598_2018_19896_MOESM1_ESM.pdf]
